# Driving the Green Transition: Innovative Tyre Formulation Using Agricultural and Pyrolysed Tyres Waste

**DOI:** 10.3390/polym17172275

**Published:** 2025-08-22

**Authors:** Carlo Di Bernardo, Francesca Demichelis, Mehran Dadkhah, Debora Fino, Massimo Messori, Camilla Noè

**Affiliations:** 1 Department of Applied Science and Technology (DISAT), Politecnico di Torino, Corso Duca degli Abruzzi 24, 10129 Torino, Italy; carlo.dibernardo@polito.it (C.D.B.); francesca.demichelis@polito.it (F.D.); mehran.dadkhah@polito.it (M.D.); debora.fino@polito.it (D.F.); massimo.messori@polito.it (M.M.); 2 National Interuniversity Consortium of Materials Science and Technology (INSTM), Via G. Giusti 9, 50121 Firenze, Italy

**Keywords:** sustainability, rubber composites, bio-based filler, waste tyres, agricultural waste, LCA, LCC

## Abstract

The rubber industry is facing increasing pressure to adopt sustainable practices due to environmental concerns associated with the use of non-renewable resources and the growing accumulation of waste tyres and agricultural byproducts. This study explores the potential of partially replacing conventional carbon black (CB) with sustainable alternatives derived from agricultural waste (wine by-products) and pyrolysed waste tyres in natural rubber/styrene-butadiene rubber (NR/SBR) composites for tyre applications. A series of NR/SBR composites were formulated with varying ratios of CB to agricultural waste and pyrolysed tyre waste, while maintaining consistent levels of other additives. The resulting composites were then subjected to a comprehensive suite of analyses, including scanning electron microscopy (SEM), Brunauer–Emmett–Teller (BET) surface area measurements, Fourier transform infrared spectroscopy (FTIR), bound rubber content determination, Payne effect analysis, thermogravimetric analysis (TGA), dynamic mechanical thermal analysis (DMTA), and mechanical property testing. Furthermore, a Life Cycle Assessment (LCA) and Life Cycle Costing (LCC) analysis were conducted to evaluate the environmental and economic viability of the proposed CB replacements. The results reveal that the incorporation of agricultural waste and pyrolysed tyre waste can significantly impact the curing behaviour, mechanical properties, and thermal stability of rubber composites. Importantly, some of the formulations demonstrate comparable tensile strength, elongation at break, and hardness compared to traditional CB-filled composites. The LCA and LCC analyses further highlight the potential for substantial reductions in greenhouse gas emissions, fossil resource depletion, and overall production costs, thereby supporting the transition toward more sustainable tyre manufacturing practices.

## 1. Introduction

Rubber is a versatile material that can produce a wide range of valuable products. The continuous advancement in rubber technology promises the emergence of new products from the rubber sector. Two types of rubber dominate industrial usage: natural rubber (NR) and synthetic rubber. Natural rubber finds widespread application across industries owing to its elasticity, low hysteresis, remarkable durability, and superior toughness. Although NR is generally considered amorphous, its ability to strengthen when stretched significantly enhances its mechanical attributes, including tensile strength, tear resistance, and abrasion resistance [1].

Polymer composites are commonly used in various technical applications, including gaskets, bearings, high-pressure hoses, cables, and tyres, due to their facilitated processing, increased productivity, reduced costs, superior dynamic, mechanical, creep, and wear resistance properties [2,3]. The use of sustainable rubber compounds is growing due to environmental concerns caused by the widespread use of polymers and the need to transition to a circular economy [4]. Rubber-based materials usually have a low corrosion rate and do not require lubrication throughout their service life. Moreover, raw rubber is weak, swells in liquids, and degrades in oxygen or ozone environments [5]. To address this, adding reinforcing filler is essential for improving the properties of rubber composites to an acceptable level to meet the practical demands [6,7]. Various materials are commonly incorporated during the manufacturing process.

Carbon black (CB) is a commonly used reinforcement filler in rubber products. It has been used as a rubber reinforcing filler since the third century B.C. in China. It is added to enhance the physical features of the rubber, such as hardness, tensile strength, and tear strength [8,9]. CB is typically found in the form of aggregates. The fundamental properties that contribute to the reinforcement of rubber composites are particle size, aggregate morphology, and microstructure. Smaller grain sizes of CB are especially effective in this role. Nonetheless, CB is typically produced from non-renewable petroleum resources, contributing significantly to global warming and environmental issues [10,11,12]. It is important to note that the use of fossil fuels for CB synthesis is a significant obstacle to its sustainable use in the long term, leading to rising prices [13]. These factors have significantly impacted on the cost of rubber compounds, making it difficult for rubber industries to survive in a highly competitive market. Therefore, developing environmentally friendly materials as CB alternatives is gaining worldwide attention to reduce fossil fuel use and move towards sustainable materials [14]. Achieving sustainability involves diminishing raw material consumption, exploring reuse and recycling, and examining alternatives sourced from biomaterials.

In addition to non-renewable or fossil fuel-based pollutants, the large amount of withered farm residue left after harvesting primary crops is also a significant concern for the ecosystem. Recent research has focused on incorporating biomass made from agricultural waste materials into polymeric composites [15,16,17]. This approach offers convenience and sustainability benefits by emphasising that these biodegradable particles are produced easily from agricultural waste at zero cost. The fibres derived from this biomass have several advantages, including natural biodegradability, renewability, and bulk availability. These properties make it essential for producing biocomposites with improved physicomechanical properties [18]. The performance of biocomposites depends on various factors, including the matrix polymer, the source, size, shape, and chemical composition of the reinforcement, and the volume fraction of the filler. Manufacturing methods and the interaction between the filler and matrix also play a vital role in determining the performance of the developed bioreinforced-composites [19]. Researchers have developed technically superior rubber composites using modified natural fibre-based materials derived from short jute, bamboo, coir, sisal, oil palm, kenaf, grass, hemp, pineapple leaf [20,21], and more recently, banana stem. Compared to raw rubber compounds, these reinforced rubber composites showed improved mechanical properties [22,23].

The fast development of the car industry leads to dynamically increasing amounts of rubber waste, especially in the form of waste tyres, becoming a severe environmental problem [24]. There is a stable 3D network structure in scrap rubber, which is the most resistant to the action of microorganisms, making it challenging for the rubber to degrade naturally in the earth [25]. Moreover, waste tyres can release harmful environmental pollutants, such as heavy metals, volatile organic compounds, and particulates. It can also contaminate soil and water, posing a health risk to humans and animals. Recycling used tyres is one of the most environmentally friendly ways of reducing their impact [26]. Thermal pyrolysis is one of the main methods to recycle energy and raw materials from waste tyres, as it can convert scrap tyres into oil, gas and char products, in addition to steel, at a typical pyrolysis temperature of 500 °C [27,28]. In recent years, significant progress has been made in the sustainable management of waste tyres, including the development of more efficient recycling methods and the use of waste tyre-derived materials in various applications. For this reason, used tyres should no longer be considered hazardous waste in the world but as a source of ‘environmentally friendly’ materials that can be transformed into new tyres (retreading), energy (incineration in cement kilns, paper mills and industrial boilers) or a new group of composite materials with a wide range of applications (recycling) [29]. One of the main methods of sustainable management of used tyres is to grind them and use the rubber granulate obtained as a component or filler to manufacture new polymer composites, reducing the number of post-consumer tyres.

However, even if different CB replacements in natural or synthetic rubbers have been investigated in the literature, very few investigations evaluate their actual environmental impacts and economic costs. Moreover, the available environmental analyses primarily focus on traditional natural rubber production processes [30], raw material extraction considering the carbon footprint [31] and the cultivation or production of agricultural fillers [32]. Only one study, to the best of the authors’ knowledge, quantifies the environmental impacts of a rubber nanocomposite in which 50 wt% of CB has been substituted by cellulose nanocrystals [33]. Additionally, very few articles compare the mechanical properties of filled rubber with composites containing CB, as most research concerns the comparison between unfilled and filled rubber.

To fill this gap, this paper explores a char derived from pyrolysed tyres (Gen 1.5) and wine by-product (WPL GS) as a suitable replacement for CB in rubber composites (natural rubber/styrene butadiene rubber). Different rubber composites containing different CB replacement amounts were prepared and analysed to identify the optimal substitution concentration. This study also deeply examines the microstructures effect and chemical compatibility of Gen 1.5 and WPL GS fillers on the thermal, thermomechanical and mechanical properties of the obtained rubber composites. Finally, the environmental impact and economic analysis of the partial replacement of CB with Gen 1.5 and WPL GS was performed through Life Cycle Assessment (LCA) and Life Cycle Costing (LCC) methodologies, respectively.

## 2. Materials and Methods

### 2.1. Materials

Styrene-butadiene rubber (SBR), NR, sulphur (S), stearic acid (SA), zinc oxide (ZnO), Mild Extracted Solvate (MES) oil, and accelerator N-tert-butyl-2-benzothiazole sulphenamide (TBBS), Commercial N326 carbon black (CB) (BET area of 77 m^2^/g, average pore dimensions 27 nm) and pyrolysed tyres char (Gen1.5) were provided by Michelin S.p.A. Agricultural waste derived from wine seeds by-product (WPL GS) was provided by AgroMateriae srl and utilised as a biofiller.

### 2.2. Preparation of Rubber Composites

Different rubber composites were prepared by adding different CB-biofiller amounts in the (NR:SB, 50 wt%:50 wt%) matrix, while keeping the remaining additive’s quantity equal, as reported in Table 1. To obtain homogeneous formulations, the compounds were mixed using a laboratory mixer, Brabender at 130 °C with rotor speed set at 90 rpm for 30 min. The final rubber composite mixtures were cured using compression moulding (Gibitre) at 140 °C for 15 min. Samples were labelled following the nomenclature SX, where “S” stands for specimen and “X” can either be 0 (reference) or a number from 2 to 7, indicating the percentage of CB replacement presented in Table 1.

### 2.3. Characterisation of Tyre Composites

#### 2.3.1. Scanning Electron Microsc1opy (SEM)

The morphologies of the fillers and the fractured surface of the tensile test specimens were investigated by scanning electron microscopy SEM (Zeiss EVO 15) instrument. All the samples were covered with a 5 nm thick film of gold. Energy dispersive X-ray (EDX) spectroscopy was used to characterise the composition of the fillers.

#### 2.3.2. Brunauer–Emmett–Teller (BET)

Microstructural parameters of the fillers, including average pore size, surface area, and pore volume, were determined using the high-performance adsorption analyser (ASAP 2020 PLUS, Micromeritics^®^, Norcross, GA, USA). These measurements were conducted according to the Brunauer–Emmett–Teller (BET) theory and the Barrett-Joyner-Halenda (BJH) model. Before analysis, the fillers were degassed for 3 h at 150 °C. Nitrogen gas (N_2_) was used as a probe molecule with a surface area of 16.2 Å^2^. The experiment was conducted at a relative pressure of 0.3 and a temperature of 77 K.

#### 2.3.3. Attenuated Total Reflectance-Fourier Transform Infrared Spectroscopy (ATR-FTIR)

CB, Gen 1.5 and WPL GS were characterised by Fourier transform infrared spectroscopic analysis (FTIR) in ATR mode on TENSOR 27 FTIR spectrometer (BRUKER). Spectra were acquired in 400–4000 cm^−1^ wavelength range at 4 cm^−1^ resolution and with 32 scans.

#### 2.3.4. Bound Rubber Contents

To determine the bound rubber content (BRC) from the non-vulcanised composites the following procedure was followed. Samples of 250 g of rubber specimens were placed in toluene (40 mL) in a glass vial at room temperature for 7 days. Over this period, the solvent had not been changed, and the vials were tightly closed to avoid leakage or spilling. After 7 days, the samples were filtered over filter paper and dried overnight in the oven at 100 °C. Finally, the specimens were weighted, and the BRC was calculated by the following Equation (1):

(1) $$BRC\left(\%\right)=\left(\frac{W_{f}p-W_{f}}{W_{p}}\right)\cdot 100$$

where *W_f_* and *W_p_* are, respectively, the weights of filler and rubber in the specimens, while *W_fp_* is the weight of the specimen after the toluene extraction [34].

#### 2.3.5. Payne Effect

The Payne effect was studied by measuring the storage modulus as a function of strain amplitude using an oscillatory disc rheometer (Anton Paar, MCR702E). The test measured the storage shear modulus (G′) of filled unvulcanised rubber composites (circular samples with 25 mm diameter and 3 mm thickness) at a temperature of 25 °C and frequency (1 rad/s), with the strain amplitude varied from 0.01% to 100%. The Payne effect was quantified as the difference in storage moduli at low strain (0.01%) and high strain (100%) as shown in Equation (2) [35]:

*G′*_0_ − *G′*_100_ = Δ*G* (2)

where *G′*_0_ and *G′*_100_ are the maximum storage modulus (at the strain amplitude 0.01%) and minimum storage modulus (at the strain amplitude 100%), respectively.

#### 2.3.6. Thermogravimetric Analysis (TGA)

The changes in thermal stability of the composites were examined by thermogravimetric analysis using TGA-SDTA 851, Mettler Toledo (Columbus, OH, USA). The measurement was performed under inert atmosphere (Ar 50 μL/min) from 25 to 800 °C with a heating rate of 10 °C/min.

#### 2.3.7. Mechanical Properties

Mechanical properties of the filled rubber composites, such as tensile strength, elongation at break, and modulus, were determined using an INSTRON universal testing machine (Ulm, Germany). The test was carried out at 25 °C and a speed of 200 mm/min. The samples were cut from the vulcanised rubber sheets with a thickness of about 2 mm. The hardness, measured in Shore A, was determined using a Shore A durometer (Kern & Sohn GmbH, Balingen, Germany) based on ISO 868 [36,37]. Each measurement was repeated 3 times.

#### 2.3.8. Environmental Analysis

The environmental analysis was conducted using the Life Cycle Assessment (LCA) methodology. The study was conducted using SimaPro 9.5 software and the Ecoinvent 3.5.0 database.

The goal of the study is to compare the environmental impacts of filler-reinforced rubber composites based on the materials and ratios reported in Table 1. The fillers used to produce the reinforced rubber composites are the agro-waste (bio-fillers) and char-derived from pyrolysed tyres (char-fillers).

The scope of this study is to determine whether the increase in the percentage weight of agro-waste or char-derived tyres in the rubber composition can reduce the environmental impact of the new rubber formulation. The functional unit (FU) of the study is the amount of filler-reinforced (with agro-waste and char-derived tyres) rubber composites equal to 1 kg/d [33]. The reinforced rubber composite process was based on the data obtained at the laboratory scale, but it was scaled up at the pilot scale. The research at the laboratory scale is fundamental for understanding the technical feasibility of the process, but from an environmental perspective, the laboratory scale can provide a limited indication of the possible environmental impact of that same material or process at industrial production [38]. This decision was based on the consideration of Piccinno [38] who stated that at the laboratory scale, the energy consumption of the equipment is higher than that at the pilot scale.

In this way, the performance of the environmental study at the pilot scale avoided overestimating the energy costs.

In this study, the proportions of materials used to produce the agro-waste and char filler-reinforced rubber composite were kept the same as those tested at the laboratory scale. Since the process is based on chemical and physical reactions, scale effects were not considered. However, due to the larger amount of material used, the Brabender laboratory mixer was replaced with a Planetary mixer P 600, which has a capacity of 2500 cm^3^.

The adopted approach was from the grave (agro-by-product and tyre waste collection) to the gate (filler-reinforced rubber composite) according to Ugwu [39]. The inventory data is based on Table 2, but the data are scaled up according to the FU as reported in Table S4. The bio filler was derived from agro-waste; hence, it was considered a zero burden. The only impact associated with agro-waste was the transport on the road with a Truck Euro 5 for 10 km.

Whereas the biochar-derived tyre was produced at the laboratory-pilot scale in a fixed bed reactor (Carbolite Horizontal Folding Tube Furnace TS1 Large) at 550 °C, with a heating rate of 15 °C/min and a residence time of 30 min according to Al-Salem [40], yielding 41% biochar, 38% oil and 21% pyrogas based on weight. The high heating value of pyrogas is 25 MJ/kg according to the gas composition measured with the Micro-GC Inficon Fusion with 4 module Chassis (Table S1), which is consistent with the one reported in the literature [41]. The only impact associated with tyre waste was the transportation on the road with a Truck Euro 5 for 10 km, the consumption of the fixed bed reactor is 5.4 kWh.

The details of the considered flows taken from Ecoinvent 3.05 and the LCI are reported in Table S4.

According to Thushari et al. (2020) [42], only the direct environmental impacts of bio-filler and char-reinforced rubber composites were considered, as the environmental effects of infrastructure were excluded, given the focus on the investigated plant. The production system of bio-filler and char-reinforced rubber composites comprised both a foreground and a background system, as described by Clift et al. [43]. The foreground system directly interacted with the reference flow, whereas the background system, linked to the foreground system, included processes such as energy production and chemical supply [42]. This study was geo-contextualised in Italy, adopting the Italian energy mix. An Attributional LCA with mass allocation of multiple outputs was applied. Life Cycle Impact Assessment (LCIA) was conducted using the ReCiPe Midpoint (H) 2016 method to provide a comprehensive overview of the studied process’ impacts across various impact categories. The interpretation phase involved evaluating the resulting environmental impacts based on the contributing flows. Additionally, during the interpretation phase, potential mitigation strategies were proposed for the identified process hotspots.

#### 2.3.9. Environmental Analysis

Economic analysis has been performed through Life Cycle Costing (LCC) analysis. LCC is a systematic approach to evaluating the total cost of a product or process over its entire life cycle. It highlights the long-term cost implications of investment decisions, including acquisition, operation, maintenance, and disposal (Table 3).

The goal of the study is to evaluate and compare the cost-effectiveness of filler-reinforced rubber composites over their life cycle with that of conventional rubber, which is composed entirely of carbon black (CB). As is carried out for LCA analysis, the fillers used to produce the reinforced rubber composites are the agro-waste (bio-fillers) and char-derived end-of-life tyres (char).

The study refers to the 1 kg/d production of filler-reinforced rubber composites (Table 4). In detail, bio/char-filler reinforced rubber composites were considered according to the study of LCA (paragraph 2.8 and as reported in Table S5).

The boundaries considered in the study include raw material collection and acquisition, production, usage, and maintenance of the seven rubber composites.

The investigated configurations of the rubber composite are used in automotive components, for example, in tyres.

The identified cost items include both capital costs and operational costs. Then, the revenue is considered by selling the reinforced rubber composite. The calculation of these items is reported in Table 2.

Capital costs include the pyrolysis reactor (Carbolite Horizontal Folding Tube Furnace TS1 Large) only for producing the char derived from end-of-life tyres and for both types of reinforced rubber (agro-waste and char fillers), the mixer, Planetary mixer P 600 with a capacity of 2500 cm^3^ for mixing.

The capital cost of the pyrolysis reactor is EUR 3735.40, and the mixer is EUR 960.10. The equipment cost was normalised to the current year (2025) by adopting the coefficient CEPC referring to October 2022 (the last available).

The cost of land was considered equal to 5% of the total capital cost [44]. The tax of interest was assumed to be 2% with a 5-year amortisation [44]. The amortisation was calculated with Equation (3):

(3) $$A\left(euro\right)=C_{0}\cdot \frac{i\cdot {\left(1+i\right)}^{n}}{{\left(1+i\right)}^{n}-1}$$

where *A* is the amortisation cost. *C*_0_ means capital cost, *n* is the number of amortisation years, and *i* denotes interest tax.

Operative costs include the acquisition of raw materials, energy consumption, waste disposal, and labours. Table 5 reports the operative costs.

The agro- and tyre-wastes were derived from waste, and the only cost associated with them was the transport and collection costs. Other materials like CB, SA, ZnO, NR, SBR, and TBBS were considered, and their costs were based on the chemical price available on the Sigma-Aldrich website.

It was hypothesised that preparing bio-filler reinforced rubber requires only one worker, dedicating 0.5% of their annual working time to the task.

Revenues are based on the potential revenue derived from selling the bio/char-filler-reinforced rubber. The costs of the reinforced rubber composites are identified by considering the minimum selling price at which at least one configuration among the tested results has a positive income.

The annual profit was calculated as the difference between the revenue and the sum of the operational and amortisation costs (this last item was considered only for the first 5 years). The net present value (*NPV*) was calculated to evaluate the profitability of the tested seven configurations.

*NPV* (Equation (4)) pointed out the profitability of the tested configurations, considering a plant lifetime of 20 years, a 5% discount for the future cash flows, referring to the *NPV*. *NPV* > 0 means that the configuration is profitable.

(4) $$NPV\;\left(euro\right)=\sum_{t-1}^{T}\frac{C_{t}}{{\left(1+d\right)}^{t}}-C_{0}$$

where $C_{t}$ is the net cash flow during period *t*, $\;C_{0}$ is the initial capital investment. *t* is the plant lifetime, and *d* is the discount rate.

## 3. Results and Discussion

**Figure 1:**
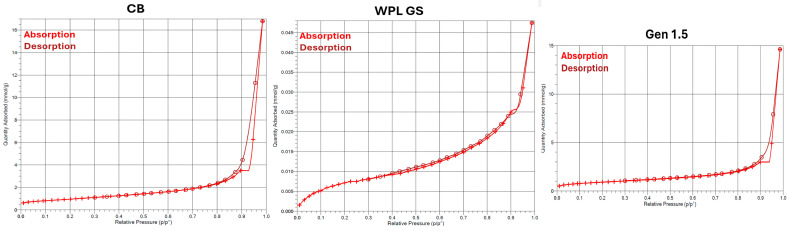
N2 adsorption isotherm of CB, WPL GS and Gen 1.5.

In Figure 1, the N_2_ absorption–desorption isotherms recorded at 77 K for the different fillers used in this work (CB, Gen 1.5, and WPL GS) are reported. As can be observed in the figure, both CB and Gen 1.5 exhibited type III adsorption isotherms, according to the IUPAC classification [46], indicating a weak interaction between the filler and the gas. While WPL GS exhibits an S-shape associated with the type II curve according to the standard classification. The II type isotherm typically refers to an unrestricted monolayer or multilayer adsorption process that can be associated with non-porous material [47].

Subsequently, the BET theory was applied to calculate the surface area of the fillers (Figure S2).

The BET surface area of CB (N326) was around 77 m^2^/g which is consistent with the values reported in the literature for other commercial CBs (N330~82 m^2^/g; N339~90 m^2^/g) [48]. The measured BET surface area of Gen 1.5~73 m^2^/g was comparable with that of the CB and with values reported in other works for pyrolysed tyres (85 m^2^/g) [49,50]. The slight difference between CB and Gen 1.5 can be attributed to the presence of carbonaceous deposits from polymer decomposition during the pyrolysis, which reduces the surface roughness of Gen 1.5 and results in a smoother surface, leading to a lower surface area than CB [51]. The BET area calculated for the WPL GS was significantly lower than the others (0.6 m^2^/g); however, this low BET surface area is consistent with the values reported for conventional agricultural agro-waste (e.g., Rice staw~4.7 m^2^/g) [52].

The BJH model was applied to investigate the pore size distribution, since it plays a crucial role in the rubber reinforcement capability [53,54]. From the BJH model reported in Figure S3, it appears clear that both CB and Gen 1.5 possess mesoporous size pores with an average pore size values are 27.6 and 30.4 nm, respectively, while WPL GS show approximately no porosity in the mesoporous range and only a small quantity of pores in the range 2–15 nm.

**Figure 2:**
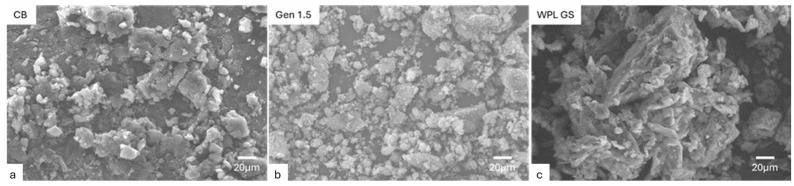
SEM images of fillers (**a**) CB, (**b**) Gen 1.5, and (**c**) WPL GS.

To further investigate the morphology of the fillers, SEM was used, and the obtained pictures are reported in Figure 2. CB shows large agglomerates with a 10–20 μm diameter, like what was observed in Ge 1.5. While WPL GS presents larger particle sizes in the range of 50–70 μm.

The elemental compositions of CB, Gen 1.5, and WPL GS were determined by performing an SEM-EDS analysis (Table S2). The results obtained from SEM-EDS analysis reveal differences in the elemental compositions of the fillers. The analysis performed on CB indicated a significant presence of carbon (C) and oxygen (O) elements: C = 98 wt.% and O = 1 wt.%, respectively. Gen 1.5 filler instead contains a slightly lower quantity of carbon (81 wt.%) and a higher quantity of O (4.5 wt.%) combined with traces of silicon (Si), sulphur (S) and zinc (Zn) which can be attributed to oxidised products or salts commonly present in pyrolysed tyres [55]. Instead, the WPL GS (filler derived from wine seeds by-product) contains only a small amount of C (28 wt%) and high quantity of oxygen (53 wt%), phosphorus (P), potassium (K), Si and S, which indicate the presence of oxides within the mixture commonly found in wine seeds [56]. The presence of those elements may potentially influence the mechanical performances of the composites [57].

**Figure 3:**
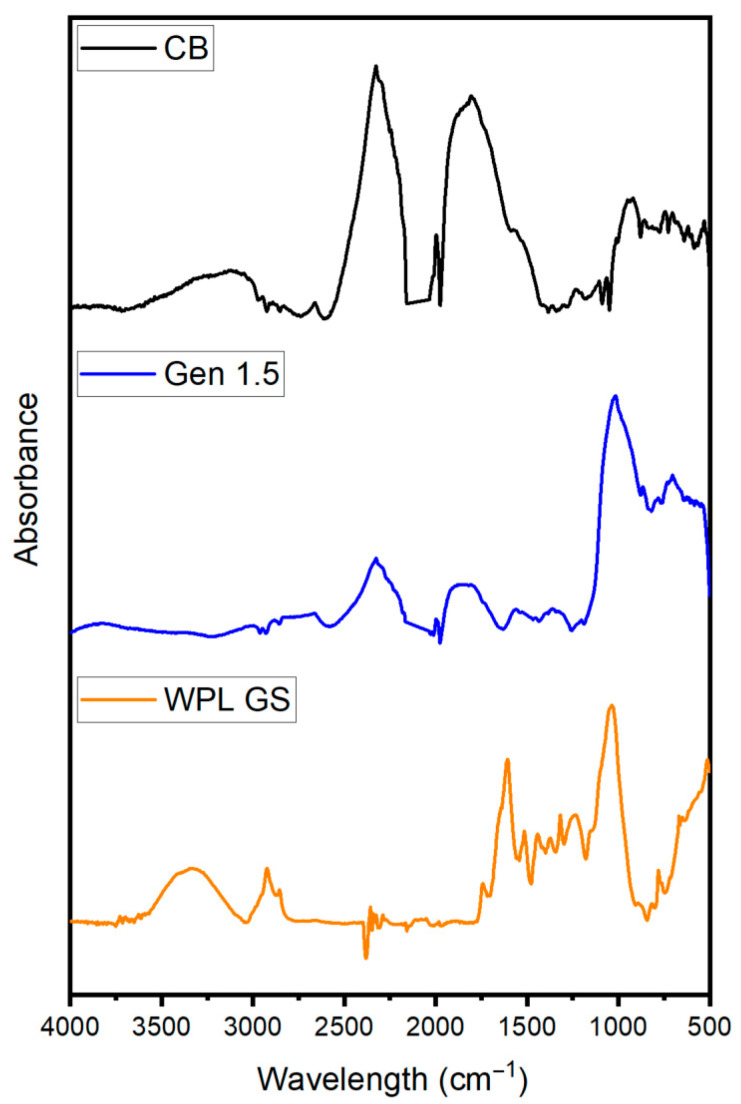
ATR-FTIR analysis of CB, Gen 1.5 and WPL GS.

To further investigate the chemical composition of the filler, ATR-FTIR spectroscopy was used. In Figure 3, the spectra of CB are compared with those of Gen 1.5 and WPL GS. The assignment of specific peaks was fundamental for identifying the surface functional groups involved in interactions with the rubber matrix. The main peak bands were assigned according to IR tables reported in the literature [58,59]. The broad bands at 3116–3030 cm^−1^ were attributed to O–H stretching of hydroxyl groups, WPL GS also present an O-H stretching band at 3326 cm^−1^ followed by two sharp peaks at 2920–2850 cm^−1^ corresponding to C-H stretching bands of unsaturated hydrocarbons. Contrarily, peaks of C-H(sp^3^) stretching bands are not present in CB and Gen 1.5, while the O-H band could be identified solely in CB sample. In the carbonyl region, characteristic peaks could be identified, which highlighted similar functional groups for CB and Gen 1.5 with anhydride peaks frequency found at 1811 cm^−1^ [60]. The band observed at 1737 cm^−1^ in WPL GS was assigned to C=O stretching related to ester moieties of tannic acid, confirmed also by the band at 1438 cm^−1^ characteristic of C–OH bending of phenols. Additional evidence of oxygenated functionalities in WPL GS (Figure 1), including phenols, ether and alcohols, came from peaks at 1373 cm^−1^ (O–H bending of phenols), 1317 cm^−1^ (C–O–C aromatic esters), 1240 cm^−1^ (C–O–C of alkyl aryl ether), likely due to the presence of lignin in the mixture and 1033 cm^−1^ (C–OH stretching) of cellulose. In CB and Gen 1.5, bands associated with identical oxygenated groups could be found at 1232 cm^−1^ (C–O–C alkyl aryl ether), 1100 cm^−1^ (aliphatic ether) and 1068–1027 cm^−1^ (C–OH stretching). Eventually, the remaining peaks in the region 900–700 cm^−1^ were assigned to C=C–H bending bands of aromatic compounds.

### 3.1. Characterisation of the Uncured Composites

- *Bound rubber test*

The term bound rubber refers to the phenomenon exhibited in reinforced rubber composites, where the polymer network is tightly bound to the filler particle nearby its surface. During the reinforcement process, the filler particles intercalate into the rubber network, establishing interactions of either physical or chemical nature at the polymer-particle interface. The strength of interactions varies according to the filler activity to the matrix, having important consequences on the reinforcement [61]. Quantifying the amount of bound rubber, therefore, gives an estimation of the network-filler compatibility and is the most direct characterisation of a rubber composite reinforcement. Although plenty of methods have been reported in the literature that employ advanced techniques, such as NMR [62] and rheology [63], swelling methods are still preferred since they do not require any special equipment. These are extraction methods based on the fact that organic solvents cannot extract bound rubber to the same extent as bulk rubber.

Similarly, to quantify the degree of interaction rubber/filler of the obtained composites, a swelling method was used [34,64].

**Figure 4:**
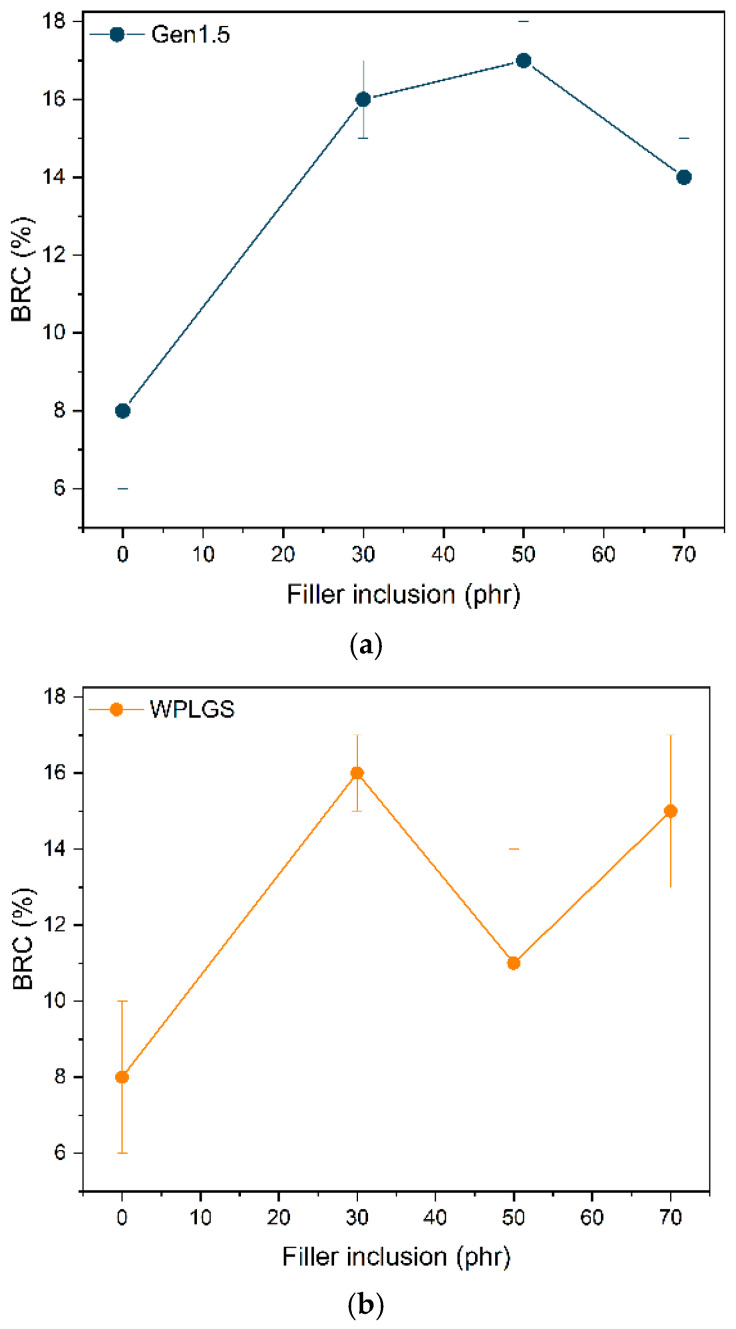
BRC (%) for different (**a**) Gen 1.5 and (**b**) BRC(%) Gen1.5 and WPLGS phr composites, respectively.

As it can be observed from the graph reported in Figure 4, the BRC of Gen1.5 composites is higher than the CB 100 phr in all cases, suggesting a good interaction of this filler towards the rubber matrix. Gen1.5 can be compared with CB due to the similar chemical and morphological features, though suggesting a good reinforcement effect in the tyre formulation. However, increasing the amount of filler at 70 phr causes a significant decrease in BRC, showing that the inclusion of Gen 1.5 is optimal at 50 phr. Evidently, the reinforcement proved to be less effective for the bio-filler, for which there can be seen a decrease in BCR with more than 30 phr of WPL GS, going from 16% to 7%. The BRC reaches the maximum value with the addition of 30 phr of biofiller. At higher inclusion of WPL GS the BRC drops due to the lower filler compatibility.

- *Payne effect*

**Figure 5:**
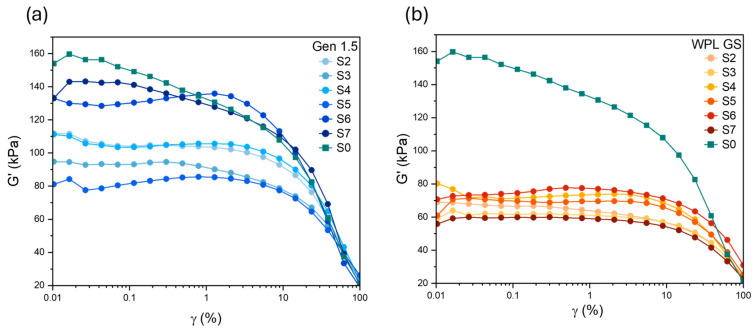
Effect of filler loading on shear storage modulus (G’) of (**a**) Gen 1.5 filled rubber composites and (**b**) WPL GS filled rubber composites.

Rubber composites contain an aggregated secondary network of filler particles held together by Van der Waals-London forces. The breakdown of this network results in a decrease in the storage modulus (G′) as strain increases, which is known as the “Payne effect” [32]. The Payne effect is a useful way to characterise the interaction between fillers and can be represented by the drop in shear modulus (△G′) from low strain (≤1%) to high strain (100%) [65]. Figure 5a,b show the changes in (G′) for rubber composites containing Gen 1.5 and WPL GS in the uncured state, respectively. From Figure 5 it can be observed that when Gen 1.5 was used to substitute CB, the Payne effect slightly decreased, probably due to its lower compatibility with the rubber matrix. Moreover, it is possible to observe a nonlinear reduction in the Payne effect with the addition of increasing filler content. While the addition of WPL GS significantly reduced the G’ values and therefore the Payne effect in all cases. Similarly to the case of Gen 1.5, different amounts of WPL GS do not result in a linear correlation with the Payne effect.

### 3.2. Characterisation of the Vulcanised Composites

- *Thermogravimetric analysis of vulcanised composites*

**Figure 6:**
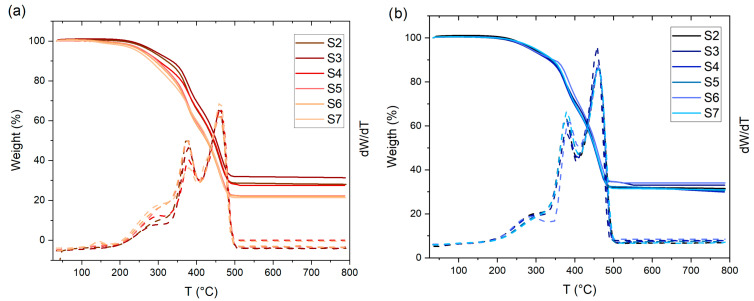
TGA and dTGA curves of filled rubber composites, (**a**) WPL GS, and (**b**) Gen 1.5.

Figure 6a,b shows the TGA of vulcanisates containing various contents of WPL GS and Gen 1.5 fillers, respectively; while in Figure S5, the pristine composite containing only CB is reported. The pristine composite (S0) shows an initial thermal decomposition at around 260 °C followed by two main degradation steps (382 °C, 460 °C), which are consistent with the values reported in the literature for tyre composites [41]. The initial small weight loss decomposition step is attributed to the volatilisation of the oil and to the other low-boiling materials present in the rubber matrix; while the main two weight-loss steps are linked to the decomposition of the natural rubber (~350 °C), and to the decomposition of the styrene-butadiene rubber (~425 °C) [66].

All the degradation temperatures and char residue % are reported in Table S5. As can be observed, the addition of WPL GS biofiller slightly reduced the char residue from 34% (S0) to 21% (S7). This result can be attributed to a higher organic content of WPL GS with respect to other fillers. While the substitution of CB with Gen 1.5 did not lead to a variation in the char residue.

Interestingly, all the composites showed the two main degradation peaks in the same temperature range of the S0, suggesting that the substitution of CB with the new fillers does not negatively affect the thermal stability of the rubber composites.

- *Mechanical properties of the vulcanised rubber composites*

**Figure 7:**
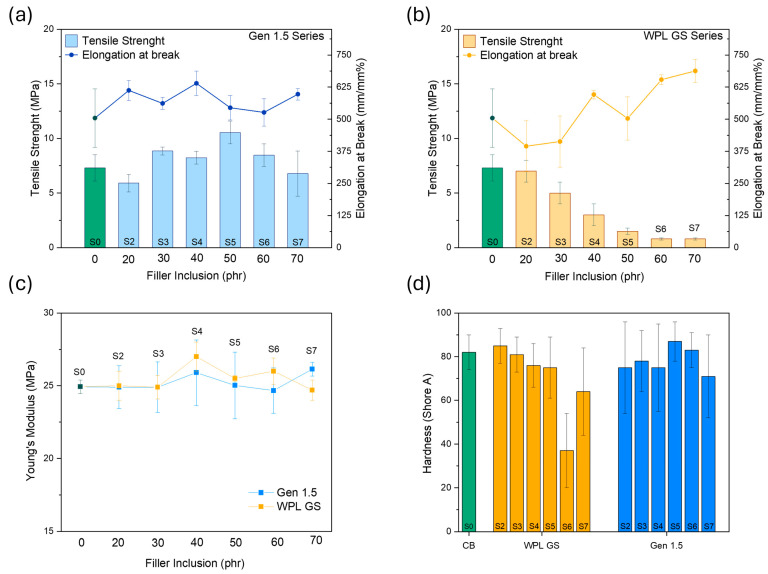
Mechanical properties of vulcanised rubber composites filled with Gen and WPL at various filler content: tensile strength and elongation at break for (**a**) Gen 1.5, (**b**) WPL GS, (**c**) Young’s modulus and (**d**) Hardness for the corresponding composites.

Figure 7a,d shows the effect of WPL and Gen 1.5 loading on changes in mechanical properties for vulcanised rubber composites. As it can be observed from Figure 7a, increasing the amount of WPL GS filler in the rubber composites led to a noticeable decrease in both tensile strength and hardness, going from 7.3 MPa–82 Shore A for the S0 (100:0, only CB) to 0.8 MPa–63 Shore A for S7 (30:70 CB: WPL). This could be explained considering the higher hydrophilicity (higher -OH groups content on the surface as observed from ATR-FTIR) of the WPL GS filler, resulting in a low compatibility between the rubber and the WPL GS fillers. These results are in good agreement with the ones obtained from the Payne effect. However, the progressive substitution of WPL GS with respect to CB has a positive effect on the material’s ductility, enhancing its elongation at break, while it does not appear to impact the composites’ stiffness negatively.

The composites with Gen 1.5 showed better rubber–filler interaction, enhancing the mechanical properties of the composites. As it can be observed in the (Figure 7a–d), the best performing formulation was the one containing 50 phr Gen 1.5, with an average tensile strength of 10.5 MPa, elongation at break of 526%, Young’s modulus of 25 MPa, and hardness of 87 (Shore-A). While the composites containing more than 50 wt% of Gen 1.5 showed a slight reduction in the mechanical performances, they still reached values comparable or even higher than the pristine one containing only CB.

**Figure 8:**
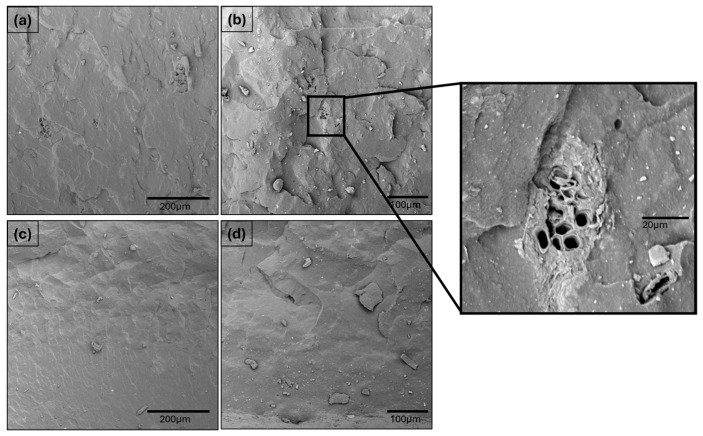
SEM micrographs of fractured surfaces of WPL GS S3 (**a**,**b**) and Gen (**c**,**d**).

Figure 8 reports the SEM images of WPL GS S3 and Gen 1.5 S3 fractured surfaces after the tensile test. As it can be observed in Figure 8a,b in the WPL GS reinforced composites, there is a huge WPL GS fractured filler possessing some internal cavities not present in the surface of the filler reported above. This indicates that during the tensile test, the fracture propagates through the filler, suggesting a good filler–matrix interaction. However, on the fractured surface of the composites, some smaller, unattached WPL GS fillers and voids can also be detected, indicating a pull-out fracture mechanism. The pull-out mechanism can usually be attributed to a weak filler–matrix compatibility, which can be explained by the presence of high-polar groups on the WPL GS filler surface, as observed previously. These results suggest a complex combination of fracture phenomena also linked to a high quantity of non-organic elements present in this agro-waste filler.

While on the fractured surface of Gen 1.5 S3 reinforced composites (Figure 8c,d), there can only be detected filler–matrix debris and matrix cracks. These fracture mechanisms suggest excellent filler–matrix compatibility; therefore, those results are in good agreement with the previous data.

### 3.3. Environmental Analysis Evaluation

The environmental assessment was performed according to the Life Cycle Assessment (LCA) methodology. This LCA aimed to compare the environmental impacts of filler-reinforced rubber composites to assess whether using fillers derived from agro-waste or char from end-of-life tyres, as alternatives to carbon black (CB), can reduce the environmental impact of rubber production. Scenario 1 (S0) represents the current commercial rubber composite without fillers, serving as the baseline scenario. Scenarios 2 to 7 (S2–S7) explore new formulations of filler-reinforced rubber composites designed to replace CB with fillers derived from agro-waste or char from end-of-life tyres.

The environmental impact assessment was conducted using ReCiPe MidPoint (H) 2016, with the functional unit defined as 1 kg/d of filler-reinforced rubber composite.

**Figure 9:**
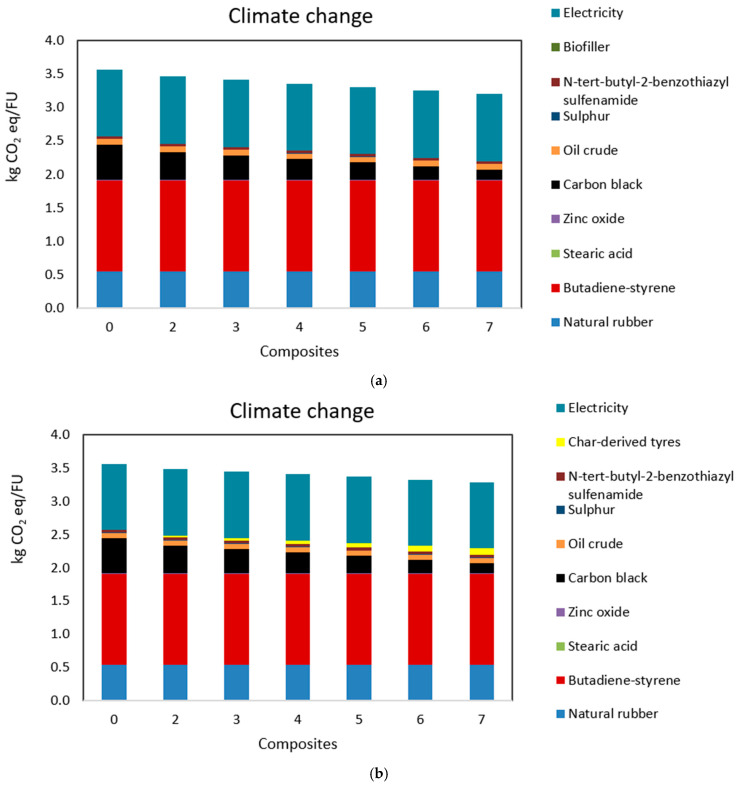
Environmental comparison of the produced composites with details about the contribution of each item of the composite. FU = 1 kg/d of filler reinforced rubber composites. In Figure (**a**), the reinforced rubber is with bio-filler, and in Figure (**b**), the reinforced rubber is char-derived end-of-life tyres.

Overall, the results (Figure 9) indicate that increasing the filler content reduces environmental impacts across all categories, as fillers progressively replace CB. This trend was detected both using bio-fillers derived from agro-waste and char derived from end-of-life tyres. This shift aligns with the goals of the Green New Deal and Mission 2 of the National Recovery Plan (PNRR), targeting a decrease in GHG emissions and net-zero emissions by 2050.

Focusing on climate change impacts, the environmental assessment shows a decrease in CO_2_ equivalent emissions from 3.6 kg CO_2_ eq/FU in S0 to 3.2 kg CO_2_ eq/FU in S7 considering bio-filler deriving from agro-waste, and 3.29 kg CO_2_ eq/FU in S7, considering as filler the char derived from end-of-life tyres.

Replacing CB with char derived from end-of-life tyres is less environmentally beneficial than replacing CB with bio-filler derived from agro-waste. This is because the environmental impact of tyre-derived char is significantly influenced by the pyrolysis process, which is an endothermic process. This means that pyrolysis requires more energy than it produces, making it an energy-demanding process. The environmental gap between the rubber reinforced with char and the pyrolysis process itself reflects the environmental cost associated with performing pyrolysis.

However, in both cases, using bio-filler and char filler, the global warming potential decreased compared to use 100% CB.

This reduction highlights the fillers’ effectiveness in lowering the carbon footprint, largely due to the decreased reliance on fossil-fuel-derived CB. These findings support the study by Luburra et al. (2022) [67], which demonstrated that substituting CB with biochar as a rubber bio-filler reduces greenhouse gas (GHG) emissions and the overall carbon footprint of rubber products. To better understand the path to achieving net zero, Figure 9a,b provides a detailed analysis of climate change impacts. It compares the composite scenarios, breaking down the contributions of individual components. Figure 9 reveals that the two most impactful elements are styrene-butadiene rubber and electricity, accounting for 38% *w*/*w* and 28% *w*/*w*, respectively. Hence, to further reduce the impact of the rubber production, the two compounds could be further investigated (to partially replace them or reduce their addition). The replacement of CB with fillers shows promise, with the climate change impact attributed to CB decreasing from 15% in S2 to 4% in S7. This confirms that incorporating bio-fillers can meaningfully reduce GHG emissions.

Beyond climate change, improvements are also observed in stratospheric ozone depletion (kg CFC11 eq) and fine particulate matter formation (kg PM2.5 eq). These trends are because CB production involves the incomplete combustion of fossil fuels, releasing volatile organic compounds (VOCs) and other pollutants. These substances contribute to tropospheric ozone formation (smog) and can indirectly affect stratospheric ozone.

In contrast, bio-fillers (derived from agricultural wastes) are often considered “carbon neutral” or “low-carbon” since the carbon in biomass was previously absorbed by plants through photosynthesis. Bio-filler production generally requires less energy and emits fewer GHGs compared to CB manufacturing. Moreover, using bio-fillers reduces dependence on fossil fuels, indirectly lowering emissions of ozone-depleting substances.

The environmental benefits extend further. The use of agricultural and tyre wastes reduces the need for their disposal, which might otherwise generate methane (a stronger GHG than CO_2_). Additionally, the shift from S0 to S7 for bio-filler reinforced rubber reveals a decline in fossil resource scarcity. CB production relies on extracting and processing fossil resources, whereas bio-fillers come from renewable agricultural by-products, cutting down fossil fuel dependency.

Regarding water consumption (m^3^), CB manufacturing often involves substantial water use for cooling and other processes. Conversely, bio-filler production, especially when using dry agricultural waste, tends to require less water. Moving from S0 to S7 consistently demonstrates environmental advantages across most impact categories. The most significant improvements are seen in global warming potential, fossil resource scarcity, and particulate matter formation. LCA analysis proved that fillers reduce reliance on fossil-based inputs and cut emissions, reinforcing their potential as a low-impact alternative.

### 3.4. Economic Analysis

The cost evaluation of the filler-reinforced rubber composite (including agro-waste or char as fillers) was performed through Life Cycle Costing (LCC) methodology.

Table 5 reports the dimensions of the equipments and the energy consumed to obtain 1 kg/d of filler-reinforced rubber composite, which means 365 kg/y of bio-fillers reinforced rubber composite.

Economic sustainability is achieved if revenues and *NPV* are higher than 0; hence, the costs of filler-reinforced rubber were evaluated from 10 €/kg to 20 €/kg according to the market trends for application of these composites in the automotive sector [68]. The capital and operational costs and profitability are reported in Table 6. In detail, Table 6 summarises the *NPV* of the configurations tested by varying the selling price of the filler-reinforced rubbers in the range of 10–20 €/kg.

Table 6 highlights that the minimum selling price required to achieve a positive *NPV*, among the range identified (10–20 €/kg) for producing the bio-filler reinforced rubber composite, is 15 €/kg, whereas for producing the char-filler reinforced rubber composite is 16 euros. At these prices, only S0 is not economically viable, as CB, which is partially replaced by varying percentages of fillers in configurations 2–7, proved to be the limiting economic factor. Notably, increasing the proportion of filler in the composite enhances economic profitability.

By comparing the *NPV* of rubber composites reinforced with fillers derived from agro-waste and tyre-derived char, the composites containing char were found to be less economically profitable. This is primarily due to the capital costs associated with purchasing a pyrolysis reactor, as well as the operational costs incurred by the significant energy requirements of the pyrolysis process and oil waste treatment.

Furthermore, bio-fillers reinforced rubber composites showed positive returns from the first year when CB was replaced by at least 50% (samples S5 to S7). In contrast, char filler-reinforced rubber composites were more expensive and had a lower *NPV* compared to CB-based rubber composites, highlighting the pyrolysis step as a key bottleneck, and as a step that required optimisation to become energy self-sustainable.

Combining LCA and LCC studies, the bio-filler reinforced rubber composite is preferable to the char-filler-reinforced rubber composite.

It is important to highlight that environmental and economic impact studies should be conducted at an industrial scale. Nevertheless, the pilot-scale analysis presented in this study provides a valuable assessment of the technical feasibility and economic sustainability of using biofillers to produce reinforced rubber.

It is crucial to note that this study did not account for the environmental and economic benefits associated with avoiding the disposal of agricultural and tyre wastes. For this research, the agricultural and tyre waste used to produce fillers was a zero burden, meaning zero impact rather than avoided impact, since it was repurposed rather than discarded. Economically, the waste was regarded exclusively in terms of collection and transportation costs, without factoring in any avoided disposal expenses.

Studies combining LCA and LCC for filler-reinforced rubber are limited. However, the results obtained in this study align with two relevant works available in the scientific literature. Specifically, Dong et al. (2022) [69] explored the integration of LCA and LCC methodologies to support the transition of the traditional rubber industry towards more sustainable practices, an approach that can be applied to assess the use of bio/recycled fillers in reinforced rubber components. Additionally, Fallis (2013) conducted a comparative sustainable study investigating the substitution of synthetic and imported rubber in passenger car tyres with domestically sourced natural rubber from the guayule plant, emphasising the potential advantages of biobased materials in tyre production.

Nonetheless, the selling price estimated in the present study falls within the range established by the global leader in advanced composites technology [68], making bio-filler-reinforced rubber composites an economically effective (without a cost increase) alternative to traditional rubber.

## 4. Conclusions

This research provides compelling evidence for the viability of utilising agricultural waste or pyrolysed tyre waste as partial replacements for CB in rubber tyre formulations. The study demonstrates that carefully designed composite formulations incorporating these waste-derived materials can achieve a balance of desirable mechanical properties, enhance thermal stability, and improve environmental performance. In particular, the addition of Gen1.5 can enhance the contact between the matrix and filler, thereby enabling improved performance. The LCA reveals significant potential to mitigate the environmental impact associated with tyre production, particularly in terms of climate change, fossil resource scarcity, and particulate matter formation. From an economic standpoint, the LCC analysis suggests that the utilisation of these alternative fillers can be cost-competitive with traditional CB-filled composites, particularly when considering the potential for revenue generation from waste valorisation.

While this study offers valuable insights into the potential of agricultural waste and pyrolysed tyre waste as sustainable tyre fillers, further research will be needed to optimise filler surface modification techniques, improve dispersion within the rubber matrix, and evaluate the long-term durability and performance of the resulting composites under real-world tyre operating conditions. Overall, this study contributes valuable knowledge to the growing field of sustainable rubber composites and paves the way for a more environmentally responsible and economically viable tyre industry.

## Figures and Tables

**Table 1:** Composites formulations CB/filler expressed in parts per hundred rubber (phr).

Materials	S0	S2	S3	S4	S5	S6	S7
**NR**	100	100	100	100	100	100	100
**SBR**	100	100	100	100	100	100	100
**SA**	2	2	2	2	2	2	2
**ZnO**	5	5	5	5	5	5	5
**CB**	100	80	70	60	50	40	30
**Oil**	40	40	40	40	40	40	40
**New filler**	0	20	30	40	50	60	70
**S**	4	4	4	4	4	4	4
**Accelerator**	6	6	6	6	6	6	6

**Table 2:** Comparative analysis of the seven composites.

			Bio-Fillers						Char-Derived Tyre					
Impact Categories	Unit	S0	S2	S3	S4	S5	S6	S7	S2	S3	S4	S5	S6	S7
Global warming	kg CO_2_ eq	3.57 × 10	3.46 × 10	3.41 × 10	3.36 × 10	3.30 × 10	3.25 × 10	3.20 × 10	3.49 × 10	3.45 × 10	3.41 × 10	3.37 × 10	3.33 × 10	3.29 × 10
Stratospheric ozone depletion	kg CFC11 eq	1.13 × 10^−6^	1.06 × 10^−6^	1.02 × 10^−6^	9.87 × 10^−7^	9.51 × 10^−7^	9.12 × 10^−7^	8.76 × 10^−7^	1.08 × 10^−6^	1.05 × 10^−6^	1.02 × 10^−6^	9.97 × 10^−7^	9.67 × 10^−7^	9.39 × 10^−7^
Ionising radiation	kBq Co-60 eq	1.68 × 10^−1^	1.65 × 10^−1^	1.63 × 10^−1^	1.62 × 10^−1^	1.60 × 10^−1^	1.59 × 10^−1^	1.57 × 10^−1^	1.68 × 10^−1^	1.68 × 10^−1^	1.69 × 10^−1^	1.69 × 10^−1^	1.69 × 10^−1^	1.70 × 10^−1^
Ozone formation, Human health	kg NOx eq	1.92 × 10^−2^	1.90 × 10^−2^	1.89 × 10^−2^	1.87 × 10^−2^	1.86 × 10^−2^	1.85 × 10^−2^	1.84 × 10^−2^	1.90 × 10^−2^	1.89 × 10^−2^	1.88 × 10^−2^	1.87 × 10^−2^	1.86 × 10^−2^	1.86 × 10^−2^
Fine particulate matter formation	kg PM2.5 eq	5.41 × 10^−3^	5.19 × 10^−3^	5.09 × 10^−3^	4.98 × 10^−3^	4.87 × 10^−3^	4.76 × 10^−3^	4.65 × 10^−3^	5.22 × 10^−3^	5.13 × 10^−3^	5.04 × 10^−3^	4.95 × 10^−3^	4.85 × 10^−3^	4.76 × 10^−3^
Ozone formation, Terrestrial ecosystems	kg NOx eq	2.67 × 10^−2^	2.65 × 10^−2^	2.64 × 10^−2^	2.63 × 10^−2^	2.61 × 10^−2^	2.60 × 10^−2^	2.59 × 10^−2^	2.65 × 10^−2^	2.64 × 10^−2^	2.64 × 10^−2^	2.63 × 10^−2^	2.62 × 10^−2^	2.61 × 10^−2^
Terrestrial acidification	kg SO_2_ eq	1.15 × 10^−2^	1.10 × 10^−2^	1.07 × 10^−2^	1.05 × 10^−2^	1.03 × 10^−2^	1.00 × 10^−2^	9.76 × 10^−3^	1.11 × 10^−2^	1.09 × 10^−2^	1.07 × 10^−2^	1.05 × 10^−2^	1.03 × 10^−2^	1.01 × 10^−2^
Freshwater eutrophication	kg P eq	1.26 × 10^−3^	1.22 × 10^−3^	1.20 × 10^−3^	1.18 × 10^−3^	1.15 × 10^−3^	1.13 × 10^−3^	1.11 × 10^−3^	1.23 × 10^−3^	1.21 × 10^−3^	1.19 × 10^−3^	1.17 × 10^−3^	1.15 × 10^−3^	1.13 × 10^−3^
Marine eutrophication	kg N eq	8.59 × 10^−5^	8.55 × 10^−5^	8.53 × 10^−5^	8.52 × 10^−5^	8.50 × 10^−5^	8.48 × 10^−5^	8.46 × 10^−5^	8.60 × 10^−5^	8.61 × 10^−5^	8.61 × 10^−5^	8.62 × 10^−5^	8.62 × 10^−5^	8.63 × 10^−5^
Terrestrial ecotoxicity	kg 1,4-DCB	6.43 × 10	6.22 × 10	6.12 × 10	6.02 × 10	5.92 × 10	5.81 × 10	5.70 × 10	6.25 × 10	6.16 × 10	6.07 × 10	5.99 × 10	5.89 × 10	5.80 × 10
Freshwater ecotoxicity	kg 1,4-DCB	6.41 × 10^−2^	6.18 × 10^−2^	6.08 × 10^−2^	5.97 × 10^−2^	5.86 × 10^−2^	5.75 × 10^−2^	5.64 × 10^−2^	6.25 × 10^−2^	6.17 × 10^−2^	6.09 × 10^−2^	6.02 × 10^−2^	5.93 × 10^−2^	5.86 × 10^−2^
Marine ecotoxicity	kg 1,4-DCB	8.79 × 10^−2^	8.49 × 10^−2^	8.34 × 10^−2^	8.19 × 10^−2^	8.05 × 10^−2^	7.89 × 10^−2^	7.74 × 10^−2^	8.57 × 10^−2^	8.46 × 10^−2^	8.36 × 10^−2^	8.25 × 10^−2^	8.14 × 10^−2^	8.03 × 10^−2^
Human carcinogenic toxicity	kg 1,4-DCB	1.34 × 10^−1^	1.31 × 10^−1^	1.30 × 10^−1^	1.28 × 10^−1^	1.27 × 10^−1^	1.26 × 10^−1^	1.24 × 10^−1^	1.32 × 10^−1^	1.31 × 10^−1^	1.30 × 10^−1^	1.29 × 10^−1^	1.28 × 10^−1^	1.27 × 10^−1^
Human non-carcinogenic toxicity	kg 1,4-DCB	1.72 × 10	1.68 × 10	1.67 × 10	1.65 × 10	1.63 × 10	1.61 × 10	1.59 × 10	1.70 × 10	1.68 × 10	1.67 × 10	1.66 × 10	1.64 × 10	1.63 × 10
Land use	m_2_a crop eq	3.38 × 10^−1^	3.33 × 10^−1^	3.31 × 10^−1^	3.29 × 10^−1^	3.26 × 10^−1^	3.24 × 10^−1^	3.22 × 10^−1^	3.34 × 10^−1^	3.32 × 10^−1^	3.30 × 10^−1^	3.28 × 10^−1^	3.26 × 10^−1^	3.24 × 10^−1^
Mineral resource scarcity	kg Cu eq	4.19 × 10^−3^	3.97 × 10^−3^	3.87 × 10^−3^	3.77 × 10^−3^	3.67 × 10^−3^	3.56 × 10^−3^	3.45 × 10^−3^	4.00 × 10^−3^	3.91 × 10^−3^	3.82 × 10^−3^	3.73 × 10^−3^	3.63 × 10^−3^	3.54 × 10^−3^
Fossil resource scarcity	kg oil eq	1.85 × 10	1.75 × 10	1.70 × 10	1.65 × 10	1.60 × 10	1.55 × 10	1.50 × 10	1.76 × 10	1.71 × 10	1.67 × 10	1.62 × 10	1.58 × 10	1.53 × 10
Water consumption	m_3_	3.73 × 10^−2^	3.72 × 10^−2^	3.71 × 10^−2^	3.70 × 10^−2^	3.70 × 10^−2^	3.69 × 10^−2^	3.68 × 10^−2^	3.77 × 10^−2^	3.79 × 10^−2^	3.80 × 10^−2^	3.82 × 10^−2^	3.84 × 10^−2^	3.86 × 10^−2^

**Table 3:** Structure of the calculation of the fixed costs.

Direct Manufacturing Cost	
**Raw materials**	C_RM_
**Waste treatment**	C_WT_
**Utilities**	C_UT_
**Operating labour**	C_OL_
**Direct supervisory and clerical labour**	0.18 C_OL_
**Maintenance and repairs**	0.06 FCI
**Operating supplies**	0.009 FCI
**Laboratory charges**	0.15 C_OL_
**Fixed Manufacturing Cost**	
**Depreciation**	0.1 FCI
**Local taxes and insurance**	0.032 FCI
**Plant overhead costs**	0.708 C_OL_ + 0.036 FCI
**General Expenses**	
**Administration cost**	0.177 C_OL_ + 0.009 FCI
**Distribution and selling costs**	0.11 COM
**Research and development**	0.05 COM
**Total costs**	
**DMC + FMC + GE**	

**Table 4:** Equipment design and energy consumption to produce 1 kg/d of filler-reinforced rubber. S is the scenario to indicate the different configurations of the composite according to the percentage of filler. The pyrolysis reactor was used only for the configuration of filler-reinforced rubbers, including char derived from end-of-life tyres as filler.

	S0	S2	S3	S4	S5	S6	S7
**Pyrolysis reactor**	3.0	3.0	3.0	3.0	3.0	3.0	3.0
**Mixer (L)**	2.50	2.50	2.50	2.50	2.50	2.50	2.50
**Daily fed (L/d)**	1.00	1.00	1.00	1.00	1.00	1.00	1.00
**Electrical energy consumed by pyrolysis reactor(kWh/d)**	2.7	2.7	2.7	2.7	2.7	2.7	2.7
**Energy consumed by the mixer (kWh/d)**	1.45	1.45	1.45	1.45	1.45	1.45	1.45
**Thermal energy consumed by the mixer (kWh/d)**	0.87	0.87	0.87	0.87	0.87	0.87	0.87
**Electrical energy consumed by the mixer (kWh/d)**	0.58	0.58	0.58	0.58	0.58	0.58	0.58

**Table 5:** Operative costs considered for the bio/recycled fillers reinforced rubber.

Operative Costs			
**Steam from boilers**	2.03	€/GJ	(Richard Turton et al., 2018) [45]
**kWh mix ITA**	0.297	€/kWh	(ARERA, 2021)
**Biomass cost collection**	22	€/t	Arpa Piemonte 2023
**Waste disposal**	19	€/t	Arpa Piemonte 2023
**Selling price of electric energy**	0.28	€/kWh	ENEL
**Selling price, thermal energy**	0.25	€/kWh	ENEL
**Stearic acid**	61.6	€/kg	Sigma-Aldrich
**ZnO**	20.8	€/kg	Sigma-Aldrich
**NR: natural rubber**	1.52	€/kg	Sigma-Aldrich
**SBR: styrene-butadiene rubber**	2.82	€/kg	Styrene Butadiene rubber (SBR) price index—businessanalytiq
**CB: carbon black**	4.0	€/kg	Sigma-Aldrich
**TBBS: N-tert-butyl-2-benzothiazyl sulphenamide**	85.39	€/kg	N-tert-Butyl-2-benzothiazolesulfenamide|95-31-8|FB33606

**Table 6:** Summary of fixed, operative costs, and profitability of the filler-reinforced rubber composite.

			Investment Costs	Amortisation	Operational Costs	Revenue	Incomes in the First 5 Years	Incomes After the First 5 Years	*NPV*
Selling price: 15 €/kg of filler-reinforced		S0	960.1	203.7	5506.2	5475.0	−234.9	−31.2	−11.8
	Bio-filler	S2	960.1	203.7	5407.7	5475.0	−136.4	67.3	25.3
		S3	960.1	203.7	5358.1	5475.0	−86.8	116.9	44.0
		S4	960.1	203.7	5308.5	5475.0	−37.2	166.5	62.7
		S5	960.1	203.7	5254.3	5475.0	17.0	220.7	83.2
		S6	960.1	203.7	5204.8	5475.0	66.5	270.2	101.8
		S7	960.1	203.7	5155.2	5475.0	116.1	319.8	120.5
	Char-filler	S2	4695.5	996.2	5767.8	5475.0	−1288.9	−292.8	−110.3
		S3	4695.5	996.2	5718.1	5475.0	−1239.3	−243.1	−91.6
		S4	4695.5	996.2	5668.5	5475.0	−1189.7	−193.5	−72.9
		S5	4695.5	996.2	5614.3	5475.0	−1135.5	−139.3	−52.5
		S6	4695.5	996.2	5564.8	5475.0	−1086.0	−89.8	−33.8
		S/	4695.5	996.2	5515.2	5475.0	−1036.4	−40.2	−15.2
Selling price: 16 €/kg of filler-reinforced		S0	960.10	203.69	5506.23	5840.00	130.08	333.77	125.79
	Bio-filler	S2	960.10	203.69	5407.74	5840.00	228.56	432.26	162.91
		S3	960.10	203.69	5358.13	5840.00	278.17	481.87	181.61
		S4	960.10	203.69	5308.53	5840.00	327.78	531.47	200.31
		S5	960.10	203.69	5254.31	5840.00	382.00	585.69	220.74
		S6	960.10	203.69	5204.80	5840.00	431.51	635.20	239.40
		S7	960.10	203.69	5155.19	5840.00	481.12	684.81	258.10
	Char-filler	S2	4695.46	996.18	5767.76	5840.00	−923.94	72.24	27.23
		S3	4695.46	996.18	5718.15	5840.00	−874.33	121.85	45.92
		S4	4695.46	996.18	5668.54	5840.00	−824.72	171.46	64.62
		S5	4695.46	996.18	5614.32	5840.00	−770.51	225.68	85.06
		S6	4695.46	996.18	5564.81	5840.00	−720.99	275.19	103.72
		S7	4695.46	996.18	5515.20	5840.00	−671.38	324.80	122.41

## Data Availability

The raw data supporting the conclusions of this article will be made available by the authors on request.

## Supplementary Materials

The following supporting information can be downloaded at: https://www.mdpi.com/article/10.3390/polym17172275/s1. Figure S1: Flowchart of the rubber composite preparation; Figure S2: BET plots of CB, WPL GS and Gen 1.5; Figure S3: BJH Adsorption plots; Figure S4: SEM images for EDS analysis of (a) CB, (b) Gen 1.5, and (c) WPL GS highlighting the location of point analysis; Figure S5: TGA and dTGA curves of S0 composite; Table S1: Pyrogas composition; Table S2: SEM–EDS analysis results of CB, Gen 1.5 and WPL GS; Table S3: Degradation temperature of different fillers and composites; Table S4: LCI based on laboratory data and scale up to FU = 1 kg; Table S5: Data taken from Ecoinvent 3.05.
