# Supplementary material for: Driving the Green Transition: Innovative Tyre Formulation Using Agricultural and Pyrolysed Tyres Waste
Source: Polymers (Basel). 2025 Aug 22;17(17):2275. doi: 10.3390/polym17172275 (PMC12430578; doi:10.3390/polym17172275)
Supplement: Supplementary file 1 [file polymers-17-02275-s001.zip › polymers-3792266-supplementary.pdf]

Supporting information - Article

Driving the Green Transition: Innovative Tyre Formulation Using Agricultural and Pyrolyzed Tyres Waste

Carlo Di Bernardo[1], Francesca Demichelis[1], Mehran Dadkhah[1], Debora Fino[1], Massimo Messori[1,2], Camilla Noè[1]*

[1] Department of Applied Science and Technology (DISAT), Politecnico di Torino, Corso Duca degli Abruzzi 24, 10129 Torino, Italy

[2] National Interuniversity Consortium of Materials Science and Technology (INSTM), Via G. Giusti 9, 50121 Firenze, Italy

* Correspondence: camilla.noe@polito.it

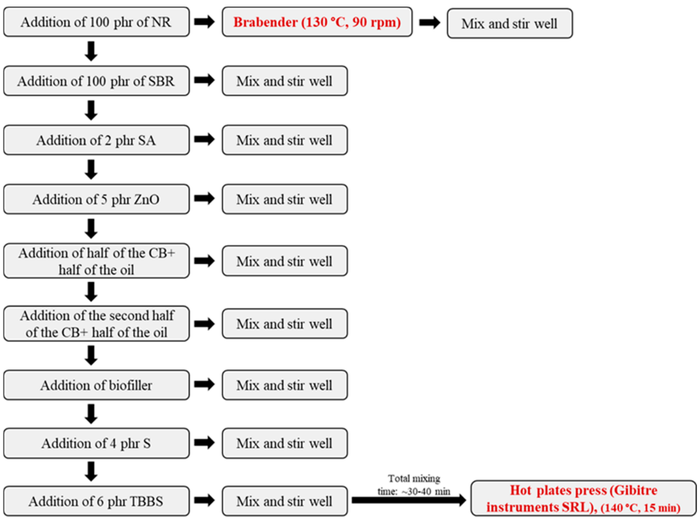

Figure S*1*: Flowchart of the rubber composite preparation.

Table S*1*: Pyrogas composition.

	**% in massa**
**H2**	**2.46**
**O2**	**0.00**
**N2**	**0.00**
**CH4**	**17.70**
**CO2**	**46.46**
**H2S**	**7.62**
**C2H6**	**7.37**
**CO**	**2.87**
**C2H4**	**6.88**
**C3**	**8.63**
**C2H2**	**0.00**

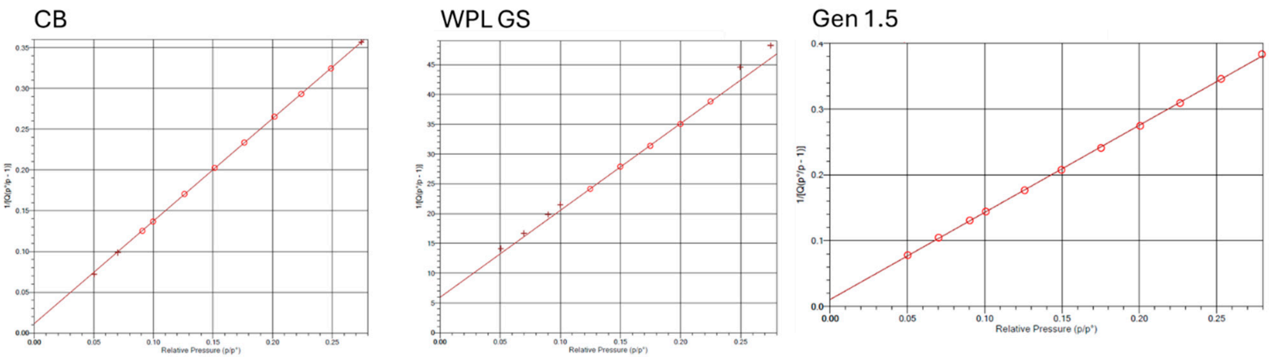

Figure S*2*: BET plots of CB, WPL GS and Gen 1.5.

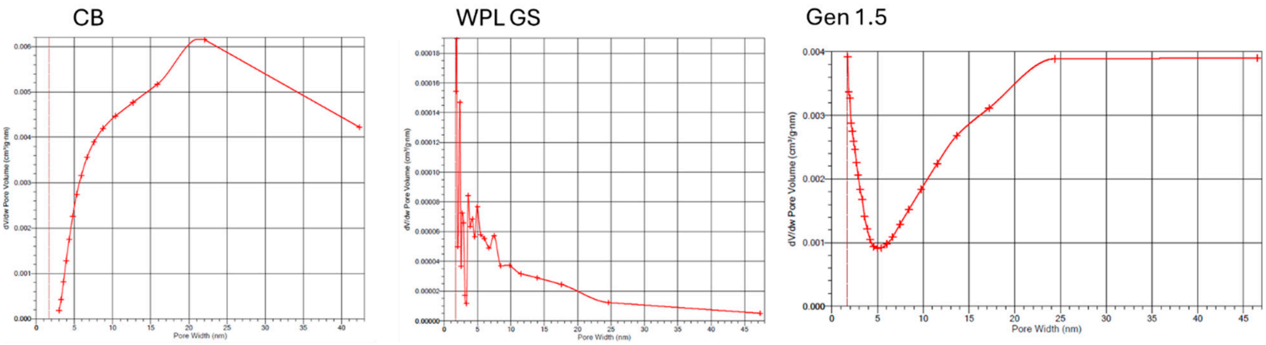

Figure S*3*: BJH Adsorption plots.

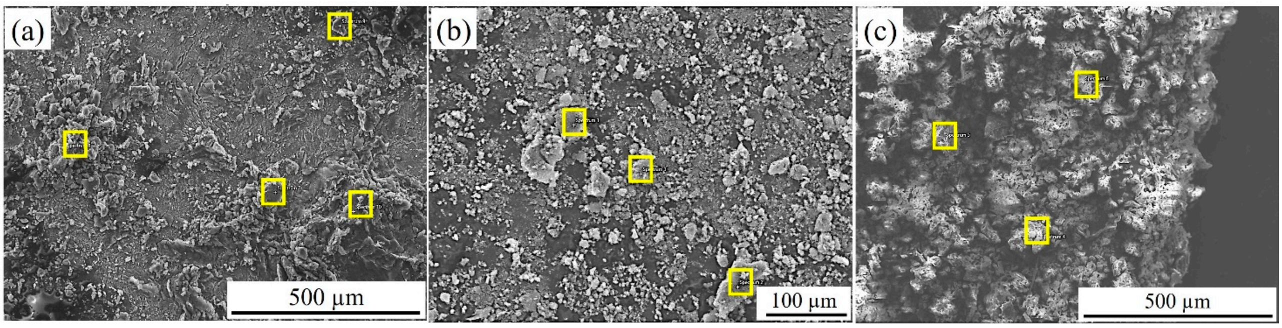

Figure S*4*: SEM images for EDS analysis of (a) CB, (b) Gen 1.5, and (c) WPL GS highlighting the location of point analysis.

Table S*2*: SEM–EDS analysis results of CB, Gen 1.5 and WPL GS.

**Element wt%**							
**Filler**	**C**	**O**	**K**	**Si**	**P**	**S**	**Zn**
CB	97.8	1.3	-	-	-	0.9	-
Gen 1.5	80.7	4.5	-	5.5	-	3.7	5.6
WPL GS	28.4	53	5.2	1.3	10.1	2	-

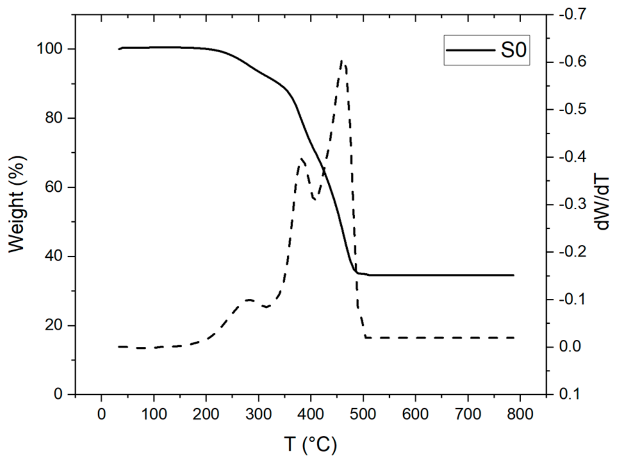

Figure S*5*: TGA and dTGA curves of S0 composite.

Table S*3*: Degradation temperature of different fillers and composites

Name	WPL GS		Gen 1.5		CB	
	T_d(°C)	Char (%)	T_d(°C)	Char (%)	T_d(°C)	Char (%)
S0	/	/	/	/	382, 460	34
S2	374, 460	31	376, 457	32		
S3	375, 461	28	378, 461	33		
S4	379, 460	27	377, 462	30		
S5	378, 460	22	376, 462	31		
S6	379, 461	21	380, 463	33		
S7	379, 460	21	377, 463	31		

***Table S4:** LCI based on laboratory data and scale up to FU = 1 kg*

**Materials (g)**	**S0**	**S2**	**S3**	**S4**	**S5**	**S6**	**S7**
	100% CB	80%CB	70%CB	60%CB	50%CB	40%CB	30%CB
		20% Filler	30% Filler	40% Filler	50% Filler	60% Filler	70% Filler
NR: natural rubber	282.12	282.83	282.83	282.83	282.12	282.83	282.83
SBR: styrene-butadiene rubber	282.12	282.83	282.83	282.83	282.12	282.83	282.83
SA: stearic acid	5.04	5.05	5.05	5.05	5.04	5.05	5.05
ZnO	12.59	12.63	12.63	12.63	12.59	12.63	12.63
CB: carbon black	282.12	224.75	196.97	169.19	141.06	111.11	83.33
Oil	110.83	111.11	111.11	111.11	110.83	111.11	111.11
Filler: agro waste/char derived end of life tyres	0.00	55.56	83.33	111.11	141.06	169.19	196.97
S	10.08	10.10	10.10	10.10	10.08	10.10	10.10
TBBS: N-tert-butyl-2-benzothiazyl sulfenamide	15.11	15.15	15.15	15.15	15.11	15.15	15.15
**Total**	1000.00	1000.00	1000.00	1000.00	1000.00	1000.00	1000.00

Table S5: Data taken from Ecoinvent 3.05

Materials	Data derived from Ecoinvent 3.05
NR: natural rubber	Seal, natural rubber based {GLO}| market for | Cut-off, S
SBR: styrene-butadiene rubber	Butadiene-styrene copolymer {GLO}| market for | Cut-off, S
SA: stearic acid	Stearic acid {GLO}| market for stearic acid | Cut-off, S
ZnO	Zinc oxide {GLO}| market for | Cut-off, S
CB: carbon black	Carbon black {GLO}| market for | Cut-off, S
Oil	Tall oil, crude {GLO}| market for tall oil, crude | Cut-off, S
Biofiller = agro waste	Is a zero burden. The only contribution considered is the transport assumed equal to 10 km.<br>Transport, freight, lorry 3.5-7.5 metric ton, euro6 {RoW}| market for transport, freight, lorry 3.5-7.5 metric ton, EURO6 | Cut-off, S
Filler: char derived from end of life tyres	Tyres are considered a zero burden. The only contribution considered is the transport assumed equal to 10 km.<br>Transport, freight, lorry 3.5-7.5 metric ton, euro6 {RoW}| market for transport, freight, lorry 3.5-7.5 metric ton, EURO6 | Cut-off, S.<br>Then the pyrolysis is performed at 550 °C, 15 °C/min for 30 min, by producing 41 % char, 38 % oil, and 21 % pyrogas at 25 MJ/kg. The pyrogas was considered as energy produced based on the Italian energy mix, while oil is considered a hazardous waste. The energy consumed to run the reactor was 2.7 kWh/d based on Italian electrical energy mix
S	Sulfur, from crude oil, consumption mix, at the refinery, elemental sulfur EU-15 S System - Copied from ELCD
TBBS: N-tert-butyl-2-benzothiazyl sulfenamide	N-tert-butyl-2-benzothiazyl sulfenamide, {GLO}| market for | Cut-off, S
